# Adaptation of *Pseudomonas aeruginosa* to the chronic phenotype by mutations in the *algTmucABD* operon in isolates from Brazilian cystic fibrosis patients

**DOI:** 10.1371/journal.pone.0208013

**Published:** 2018-11-29

**Authors:** Natália Candido Caçador, Carolina Paulino da Costa Capizzani, Lídia Alice Gomes Monteiro Marin Torres, Renata Galetti, Oana Ciofu, Ana Lúcia da Costa Darini, Niels Høiby

**Affiliations:** 1 School of Pharmaceutical Sciences of Ribeirão Preto, University of São Paulo, Ribeirão Preto, São Paulo, Brazil; 2 Department of Pediatrics of Ribeirão Preto Medical School, University of São Paulo, Ribeirão Preto, São Paulo, Brazil; 3 Department of Immunology and Microbiology, University of Copenhagen, Copenhagen, Denmark; 4 Department of Clinical Microbiology, University Hospital, Rigshospitalet, Copenhagen, Denmark; Laurentian, CANADA

## Abstract

Chronic lung infection by *Pseudomonas aeruginosa* is the leading cause of morbidity and mortality in cystic fibrosis (CF) patients. This is associated with the conversion of the non-mucoid to the mucoid phenotype. However, there is little information about the occurrence of alginate-producing *P*. *aeruginosa* in CF patients outside Europe and North America. The aim of the present study was to investigate mutations in the *algTmucABD* operon in mucoid and non-mucoid isolates from Brazilian CF patients. Twenty-seven mucoid and 37 non-mucoid isolates from 40 CF patients chronically infected by *P*. *aeruginosa* attending a CF reference center in Brazil were evaluated by sequence analysis. Mutations in *mucA* were observed in 93% of the mucoid isolates and 54% of the non-mucoid isolates. Among these non-mucoid isolates, 55% were considered revertants, since they also had mutations in *algT* (*algU*). Most isolates associated with moderate alginate production presented point mutations in *mucB* and/or *mucD*. We identified 30 mutations not previously described in the operon. In conclusion, mutations in *mucA* were the main mechanism of conversion to mucoidy, and most of the non-mucoid isolates were revertants, but the mechanism of revertance is not fully explained by changes in *algT*.

## Introduction

The onset of chronic airway infection with *Pseudomonas aeruginosa* in cystic fibrosis (CF) patients is usually preceded by a period of recurrent, intermittent colonization, most often by environmental strains [1]. During the chronic lung infection, *P*. *aeruginosa* persists and survives for decades under the challenging selective pressure imposed by an oscillating inflammatory response, continuous exposure to antibiotic therapy, and variable nutrient availability. This is mainly due to a biofilm mode of growth with contribution of virulence factors, such as alginate, and adaptive evolution mediated by genetic variation. The stressful conditions encountered by *P*. *aeruginosa* lead to mutations and these isolates are selected during the adaptation phase [1–4].

It has been shown, in the CF lung environment, these stress conditions may affect alginate production. Once the infecting bacteria become mucoid, it is very difficult to eradicate the infection due to biofilm development [5,6]. Clinically, the presence of mucoid phenotypes is associated with a poor prognosis, because of progressive deterioration of lung function. The main characteristic of the mucoid phenotype is the production of alginate, a thick mucopolysaccharide slime layer [7].

The alginate biosynthesis operon depends on the expression of the *algD* promoter, which is controlled by genes from several loci. An important feature in its regulation is AlgT (also known as AlgU or σ^22^, the sigma factor) [4]. The *algT* gene belongs to an operon with other genes, *mucABCD*. Mutations in *mucA*, *mucB* or *mucD* are important in the conversion to mucoidy, since their products have a negative regulatory effect on *algT*, while the gene product of *mucC* appears to have a controversial modulatory role, having both positive and negative regulatory functions [7,8]. Isolates with mutations in *mucA* present a highly mucoid phenotype, whereas *mucB* and *mucD* mutants are slightly mucoid and confer alginate production during growth in specific media, with alginate inducers [9,10].

In addition to alginate production, AlgT regulates a large number of stress response and virulence-associated genes, and it is involved in the regulation of motility in *P*. *aeruginosa* [1]. Therefore, when AlgT is not controlled, it is a selective advantage and must be vital for the bacteria to be able to persist in chronically infected CF patients [11]. However, it is common to observe mucoid *P*. *aeruginosa* in co-infections with revertants, non-mucoid isolates that still harbor a *mucA* mutation. The revertants can occur by secondary-site mutations in *algT* or other alginate regulatory genes [7].

The aim of the present study was to obtain a better understanding of the adaptation and genomic evolution that occurs in *P*. *aeruginosa* during chronic lung infection in CF patients. It has long been known that mutations often occur in the *algTmucABD* operon, which is associated with alginate production. Whereas previous studies have examined the isolates from CF patients in Europe and North America, the present study aimed to investigate mutations in the *algTmucABD* operon in mucoid and non-mucoid isolates from Brazilian CF patients. We identified 30 new mutations, suggesting a hot-spot region for mutation in c*algT* and supporting the hypothesis that some loci represent hot-spot regions for mutation in *mucABD*, suggesting a common selective environment in the lungs of CF patients.

## Materials and methods

### Ethics statement

The study was approved by the Research Ethics Committee of the School of Pharmaceutical Sciences of Ribeirão Preto (2010/2011) and by the hospital. Informed written consent was obtained from the patients or their relatives (for patients under the age of 18 years).

### Patients and bacterial isolates

The present study only included patients chronically infected by *P*. *aeruginosa* attending at a CF reference center in a public university hospital in Brazil (Hospital das Clínicas da Faculdade de Medicina de Ribeirão Preto—University of São Paulo) from 2011 to 2014. The age of patients ranged from 1 year old to 45 years old (median age = 14,5 years), and 18 of the patients were less than 12 years old. Pulmonary secretion cultures were conducted according to international recommendations [12], and subjected to culture every 2 or 3 months, when the patients’ clinical status was analyzed. In few of these evaluations the patients were in an acute exacerbation, but most of the isolates are from periods of stable infection.

Patients were considered chronically infected by *P*. *aeruginosa* when they fulfilled any of the following criteria: i) strain with a mucoid phenotype; ii) at least three positive cultures for *P*. *aeruginosa* among samples taken at least 1 month apart during a 6-month period [13] with persistent isolates, i.e. genetically related isolates [14]; iii) continuous presence of the microorganism in pulmonary secretions over the 6 months prior to the study [15].

When the patients were under home care, the first choice for antibiotic treatment was a combination of inhaled colistin/tobramycin and oral ciprofloxacin. When they were hospitalized, a combination of ceftazidime and amikacin was administered. In cases of resistance or presence of other non-fermenting Gram-negative bacteria in the pulmonary secretion, meropenem/imipenem and amikacin were used.

*P*. *aeruginosa* clinical isolates were streaked on Chocolate agar plates and Blue agar plates, a selective medium for Gram-negative rods (Content: 10 g peptone, 5 g yeast extract, 5 g NaCl, 1 mL maranil 5% [wt/vol] 2 mL Na_2_S_2_O_3_ 50% [wt/vol], 10 mL bromothymolblue 1% [wt/vol], 27 mL lactose 33% [wt/vol], 1.2 mL glucose 33% [wt/vol], 10 g Bacto agar, 1 L ion-exchanged tap water, pH 7.7–7.8, SSI Diagnostica, Hillerød, Denmark) [16]. One single colony of each isolate was directly deposited on a matrix-assisted laser desorption/ionization-time of flight mass spectrometry (MALDI-TOF MS) target plate (Bruker Daltonics GmbH, Bremen, Germany) for species identification [17].

### Genotyping by pulsed-field gel electrophoresis

All isolates were typed by pulsed-field gel electrophoresis (PFGE) as described previously, using *Spe*I restriction enzyme (Thermo Scientific, Waltham, MA, USA) [18]. After PFGE, using the CHEF DRIII apparatus (Bio-Rad, Hercules, CA, USA), the restriction patterns were visualized by ethidium bromide staining and photographed (AlphaImager, Alpha Innotech, San Leandro, CA, USA). The clonal relatedness of the isolates was confirmed according to Tenover et al. [19]. Isolates with pulsotypes that differed from each other by two to three bands were considered clonally related, whereas isolates with pulsotypes that differed by more than three bands were considered different strains.

### Investigation of alginate morphotypes

The screen for alginate morphotypes of the isolates was first determined on Chocolate or Blue agar plates. Afterwards, the alginate production was assessed on LB (Luria-Bertani, BD Difco, Franklin Lakes, NJ, USA) and PIA (*Pseudomonas* isolation agar, BD Difco) media, which contains a strong alginate inducer. Based on their phenotypes on PIA and LB agar, the isolates were classified as: type I: mucoid on both media; type II: mucoid on PIA but non-mucoid on LB; type III: non-mucoid on both; and type IV: very slight but detectable mucoid on both plates after 4–7 days incubation [7,11].

### Sequence analysis of the *algTmucABD* operon

DNA extraction was carried out according to the manufacturer using a commercial DNA isolation kit (Gentra Puregene, Qiagen, Hilden, Germany). Primers previously described were used for PCR amplification and sequencing of the *algT*, *mucA*, *mucB* and *mucD* genes [7]. PCR amplification of the *algTmucABD* operon was carried out using DYNAzyme EXT DNA polymerase (Thermo Scientific, Waltham, MA, USA). After purification (Wizard Genomic DNA Purification Kit, Promega, Madison, WI, USA), the PCR products were sequenced on a Macrogen automatic DNA sequencer ABI3700. The sequencing results were compared with the strain PAO1 sequence (http://www.pseudomonas.com) using ChromasPro version 1.7.6 (Technelysium Pty Ltda, Australia), in order to determine the occurrence of sequence variants within the operon. Synonymous substitutions (silent mutations) were not presented in the present article.

Since mutations in *mucB* and *mucD* lead to slightly mucoid morphotype, sequence alterations in these genes were investigated only in isolates classified as type IV alginate production.

## Results

### Genome macrorestriction analysis

Sixty-four *P*. *aeruginosa* isolates were selected in the period of study, 27 mucoid and 37 non-mucoid. Forty-eight paired mucoid and non-mucoid isolates from 24 CF patients with chronic *P*. *aeruginosa* lung infection were selected. The pairs of mucoid and non-mucoid isolates had similar PFGE patterns and were therefore considered clonally related. In addition to the 48 pairs of mucoid/non-mucoid isolates, we also included *P*. *aeruginosa* isolates from 16 chronically infected CF patients that showed only one phenotype (13 patients presented only non-mucoid isolates and 3 patients presented only mucoid isolates).

PFGE showed that most of the patients harbored isolates belonging to different pulsotypes. The exceptions were: six presented a sort of the same three pulsotypes; two patients presented clone A; two, clone B; and two, clone E (see S1 Table).

### Distribution on different alginate morphotypes

**Fig 1:**
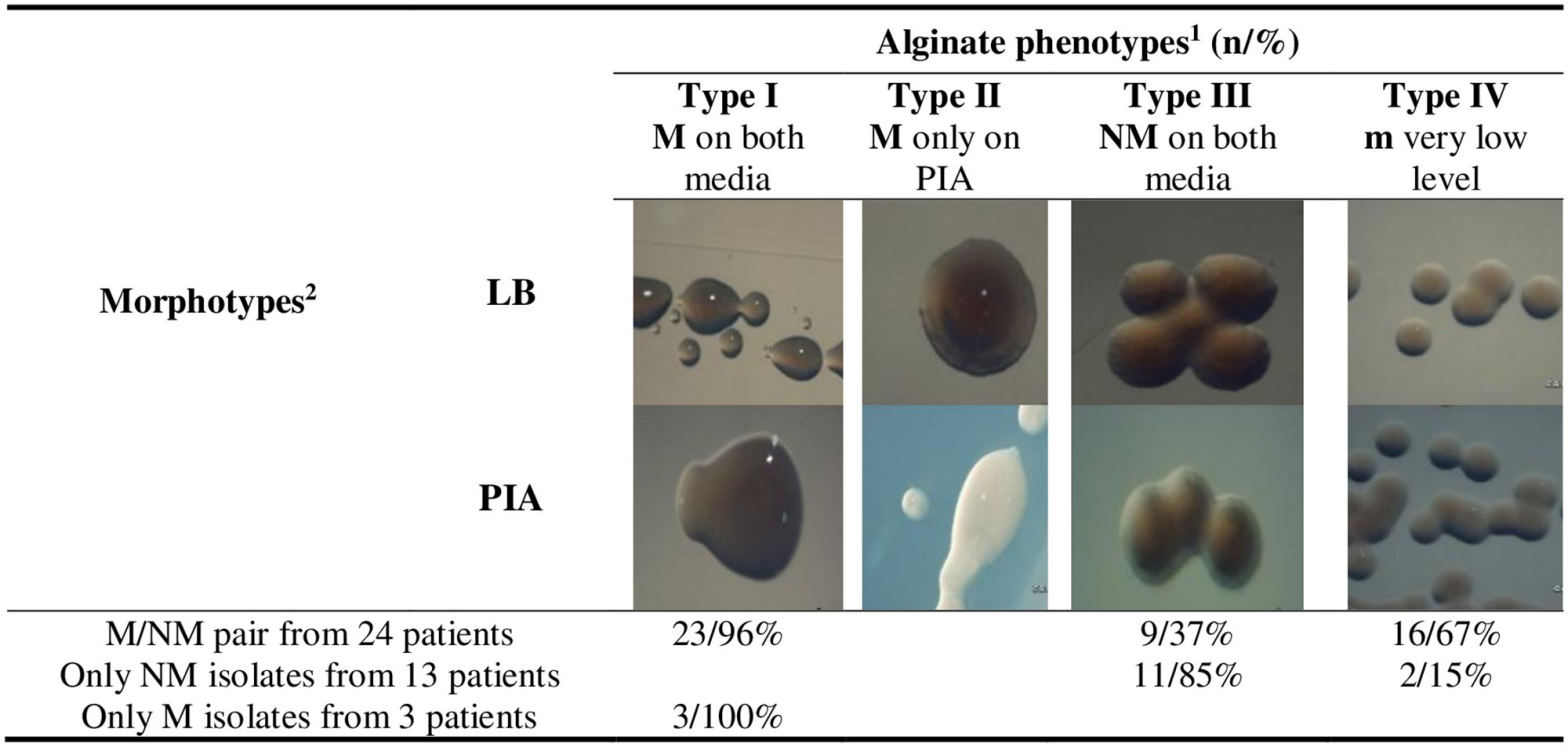
Different alginate morphotypes of *P*. *aeruginosa* isolates on two culture media LB and PIA. ^1^ M, mucoid; NM, non-mucoid; n, number of isolates; m, very low level but detectable alginate production on both media after prolonged incubation (4 to 7 days). ^2^ LB (Luria Bertani) and PIA (*Pseudomonas* Isolation Agar), after incubation at 37 °C. The pictures were obtained using the Olympus SZ61 stereo-microscope, with final magnification 45 X.

Considering the 48 paired mucoid and non-mucoid isolates, it was observed that 96% of the mucoid isolates maintained their phenotypes after culture on PIA and LB media, showing type I alginate production, whereas only 37% of the non-mucoid isolates showed type III alginate production. None of the isolates showed type II alginate morphotype. Nevertheless, one mucoid isolate (4%), and fifteen isolates (63%) that were first considered non-mucoid (63%), presented small but detectable mucoidy after a prolonged period of incubation (7 days), corresponding to type IV alginate production (see alginate morphotypes in Fig 1).

Among the isolates from 13 chronically infected CF patients with only non-mucoid *P*. *aeruginosa*, eleven isolates (85%) maintained their phenotypes on PIA and LB, showing type III alginate production, while two isolates (15%) showed type IV alginate morphotype. Among the isolates from the 3 CF patients with only mucoid *P*. *aeruginosa*, all three isolates were classified as type I.

### Mutations in the *algTmucABD* operon

Among the 64 *P*. *aeruginosa* isolates, 27 mucoid and 37 non-mucoid, for which the DNA sequence analysis of *mucA* was carried out, 93% (25) of the mucoid isolates and 54% (20) of the non-mucoid isolates had mutations in *mucA*. The mutations observed in the isolates are presented in Table 2. The most common mutation found in these Brazilian isolates was the *mucA22* mutation, known as a deletion of a G residue in a string of five G residues located between positions 429–433 of the *mucA* coding region (ΔG430 in Table 1). The majority of the isolates presented insertions or deletions that lead to frameshifts and/or premature stop codons.

**Table 1:** Mutations and the changes determined in the sequence of *mucA* in mucoid (M) and non-mucoid (NM) *P*. *aeruginosa* isolates from 40 CF patients.

Mutations in *mucA*	Sequence change	M (27^#^)	NM (37^#^)
C32T*	Ser/Phe	1	
C35G^1^^,^^2^	Ala/Gly	1	1
T45G	Asp/Glu	1	1
C137T*	Ala/Val	1	1
ΔG168	Frameshift, stop codon 283	1	
Insertion C at 238	Frameshift, stop codon 478	1	1
ΔC310	Frameshift, stop codon 385	1	1
ΔC347	Frameshift, stop codon 385	1	1
C349T*	Stop codon	2	1
C352T*	Stop codon	1	
ΔG357	Frameshift, stop codon 385	1	1
C364T^1^^,^^2^	Pro/Ser	1	1
ΔC365	Frameshift, stop codon 385	1	1
C367T^1^^,^^2^^,^ *	Stop codon	1	1
C382T*	Stop codon	1	1
G409T	Gly/Cys		1
C424T*	Stop codon	1	
ΔG430*	Frameshift, stop codon 439	6	6
ΔA437	Frameshift, stop codon 439	1	1
ΔA568	Frameshift	1	
ΔA578	Frameshift	1	1
Insertion GC at 583	Stop codon/Ala	2	2

^(1–2)^: Isolates with more than one mutation in *mucA*.

^(^*^):^ mutations that have been previously described.

^#^ number of mucoid (M) and non-mucoid (NM) isolates

**Table 2:** Mutations and the changes determined in the sequence of *mucB* and *mucD* in 18 *P*. *aeruginosa* isolates classified as type IV alginate production.

Mutations	Sequence change	M (1^#^)	NM (17^#^)
**Mutations in *mucB***			
Insertion G at 41^1^	Frameshift		1
TCC47GAA^1^	Frameshift		1
ΔC56^1^	Thr/His		1
G68A^1^	Asp/Asn		1
C338T*	Ser/Phe		1
C611G^2^	Ala/Gly		1
A631G^2^^,^ *	Thr/Ala	1	3
C643T	Arg/Cys		1
ΔT733	Frameshift, stop codon 868		1
**Mutations in *mucD***			
A409G^1^^,^ *	Ile/Val		7
C1310G^2^^,^^3^	Thr/Ser	1	1
G1321A^1^^,^^2^^,^^3^^,^ *	Val/Ile	1	4

^(1–4)^ Isolates with more than one mutation in *mucB* or *mucD*.

^(^*^):^ mutations that have been previously described

^#^ number of mucoid (M) and non-mucoid (NM) isolates

Four isolates (paired mucoid and non-mucoid) from two patients, which harbored the same clone (A, see S1 Table), also presented the same type of mutation in *mucA* (insertion GC at 583). In addition, the non-mucoid isolates also had the same mutation in *mucD* (A409G) (Table 2).

The sequence changes observed in *mucB* and *mucD* in the 18 isolates classified as type IV alginate production are presented in Table 2. Most of the isolates had point mutations leading to amino acid changes in MucB and/or MucD. One isolate showed four different mutations in *mucB*, including deletion, insertion and amino acid changes, located at 41–56 bp (Table 2 and S1 Table).

Among the 18 isolates (type IV), only 4 did not show simultaneous mutations in *mucA*. One isolate was the one previously mentioned with four mutations in *mucB* (see S1 Table); another isolate showed a deletion, ΔT733, leading to a frameshift and stop codon at 868 bp (*mucB* has 951 bp). These mutations have not been described before, to the best of the authors’ knowledge. The other two isolates showed point mutation in *mucB* (A631G) and *mucD* (A409G), resulting in amino acid changes. None of these four isolates presented mutations in *algT*.

The *algT* mutation types are presented in Table 3. Among the 20 non-mucoid isolates with mutations in *mucA*, 11 had mutations in *algT*, representing 55% of non-mucoid revertants. Among all the mucoid isolates investigated, only one showed an A/G transition at position 11 bp, resulting in an amino acid change.

**Table 3:** Mutations and the determined sequence changes in *algT* in mucoid (M) and non-mucoid (NM) *P*. *aeruginosa* isolates from 40 CF patients.

Mutations in *algT*	Sequence change	M (27^#^)	NM (37^#^)
A11G	Gln/Arg	1	
G49A	Gly/Arg		1
A86G*	Tyr/Cys		3
C133T	Stop codon		1
Insertion 9bp at 138*	Frameshift, stop codon 589		2
ΔG157	Frameshift, stop codon 295		1
A176G	Tyr/Cys		1
A508G	Thr/Ala		1
C528A	Phe/Leu		1
ΔC556	Frameshift		1

^(^*^):^ mutation that has been previously described.

^#^ number of mucoid (M) and non-mucoid (NM) isolates

## Discussion

Alginate-producing *P*. *aeruginosa* is the leading cause of persistence of biofilm-growing *P*. *aeruginosa* in the respiratory tract of CF patients, which is responsible for the high index of morbidity and mortality. The growth of *P*. *aeruginosa* colonies with mucoid phenotype is a marker of chronic biofilm infection in the lungs of these patients [20]. Nothing is known about the adaptation of *P*. *aeruginosa* to the chronic phenotype in CF patients in Latin America. The present study aimed to be the first in Brazil to analyze the mutations in the *algTmucABD* operon, which includes the most important regulators in alginate synthesis.

Conversion from non-mucoid to mucoid phenotype is related to mutations in the *mucA* gene that encodes the anti-sigma factor for AlgT [8]. The majority of the mutations seen in *mucA* in the present study probably result in loss of function of the gene products, since they lead to frameshifts and/or premature stop codons. The most common mutations in *mucA* (C/T transitions and the *mucA*22 mutation, see Table 1) in the present study were also reported in mucoid isolates from Australia, Europe, and the United States, and could also be associated with three dominant clones in Scandinavia [7,21–26]. In Brazil, isolates belonging to the same clone showed the same mutations in *mucA* and *mucD*, suggesting that a cross-infection might have occurred between the two patients after the phase of *P*. *aeruginosa* adaptation to CF lungs.

The mutations found in the present study in isolates with wild-type *mucA* and type IV alginate production suggest that the transcription of the alginate operon is increased after a long period of incubation, due to loss of function of MucB and MucD (negative regulators) caused by sequence changes in the genes. The most common single-point changes found in *mucB* and *mucD* (see Table 2) were previously described in European isolates [7,21]. As Ciofu et al. mentioned in their article, *P*. *aeruginosa* isolates with mutations in these genes might be misinterpreted as non-mucoid in the clinical microbiological laboratory [7].

Some other sequence alterations observed in the *mucABD* genes in our isolates have been previously reported and are marked in the tables, although several mutations are described here for the first time. Our findings support the hypothesis that these loci represent hot-spot regions for mutation in *mucABD*.

Among all of the 27 mucoid isolates analyzed in the present study, only one showed a transition (A11G) that resulted in an amino acid change. Since this mutation did not affect the type I alginate phenotype of the isolate, it may suggest that this mutation has no effect in the functionality of AlgT.

The mucoid phenotype is unstable, and it seems that different pathways can be involved in the reversion of mucoid to non-mucoid morphotypes [8]. Sequence analysis of the *algTmucABD* operon in *P*. *aeruginosa* isolates from Brazilian chronically infected CF patients showed that the majority (54%) of the non-mucoid isolates are revertants from the mucoid phenotype, since they presented sequence alterations in *mucA*. In our isolates, it was found that secondary-site mutations in *algT* were responsible for 55% of the non-mucoid revertants. This percentage was higher than for Scandinavian isolates, which showed only 30% of revertants due to inactivation of *algT* [7]. Nevertheless, experiments with spontaneous revertants isolated *in vitro* have shown higher percentages of inactivation of *algT*: 67% [11], 86% [7] and 90% [8].

In the present study, we found eight novel *algT* mutations (Table 3). Interestingly, one of the known mutation, a 9 bp insertion at position 138 in *algT* found in 2 Brazilian isolates, was also described in 3 Scandinavian CF isolates and in *in vitro* isolates, especially after aerobic growth, suggesting this as a possible hot-spot region [7]. Another mutation, an A/G transition at position 86 bp, was also found in *in vitro* isolates from Denmark and in non-mucoid revertants from a study in the U.S. [7,27]. It has been shown that *algT* revertants become motile, and this has been thought to be an adaptive measure to allow *P*. *aeruginosa* to move towards areas with more oxygen [28]. Another study also showed that revertant strains with non-functional AlgT present higher virulence factor production. This is to be expected, since the cells use a lot of energy to produce alginate, and AlgT is directly and indirectly involved in the regulation of virulence and motility in *P*. *aeruginosa* [1,29]. However, it has been suggested that *algT* mutants would be less accomplished in the CF lung environment and would be probably destroyed by the host’s immune response. That is because AlgT plays an important role in a variety of physiological and stress defense functions, such as heat shock, osmotic protection, protection against reactive oxygen species, repression of flagellum biosynthesis, and *algB* and *algR* work together as an important regulator of alginate induction by environmental factors (such as cell-wall-inhibitory antibiotics)[7].

AlgT regulates the *algB*, *algR*, *algD* and *armZ* operons. Thus, alternative pathways could explain the revertance to non-mucoid phenotype in the other isolates [8]. Additionally, investigation of alginate production in LB and PIA seems very useful for predicting mutations in the *algB* and *algR* genes, since the PIA medium contains triclosan, which is a strong alginate inducer through a mechanism dependent on *algT*, *algB* and *algR* [7, 29]. Therefore, type III alginate production (no alginate production) on PIA plates might characterize mutations in one of these genes. In the present study, 7 (19%) non-mucoid revertants showed type III alginate phenotype and intact *algT*; subsequently, the results could be explained by sequence changes in *algB* and *algR*.

The results of the present study showed that even under different conditions, such as patient ages, climate, and geographical distance, isolates from CF centers in Brazil, Argentina, North America and Europe have presented similar mutations in the *algTmucABD* operon. This probably reflects a common selective environment in the lungs of CF patients; it may be the inflammatory response, which does not kill alginate-containing *P*. *aeruginosa* biofilms [30].

In conclusion, the present study found that mutations in *mucA* are the main mechanism of conversion to mucoidy, with *mucA*22 as the most common mutation. Another finding was that the non-mucoid Brazilian CF isolates are revertants from the mucoid isolates, but the mechanism of revertance is not totally explained by *algT*.

## Supporting information

S1 Table General data of patients and bacterial isolates. M: mucoid, NM: non-mucoid, age at first pulmonary secretion sample, gender M: male, F: female, ^#^ number of mucoid and non-mucoid isolates, (underlined alginate phenotype), the only isolate that the phenotype on PIA did not match that on Blue/Chocolate agar; (*) mutations that have been previously described.
(DOCX)
