# Supplementary material for: Adaptation of Pseudomonas aeruginosa to the chronic phenotype by mutations in the algTmucABD operon in isolates from Brazilian cystic fibrosis patients
Source: PLoS One. 2018 Nov 29;13(11):e0208013. doi: 10.1371/journal.pone.0208013 (PMC6264809; doi:10.1371/journal.pone.0208013)
Supplement: S1 Table — M: mucoid, NM: non-mucoid, age at first pulmonary secretion sample, gender M: male, F: female, # number of mucoid and non-mucoid isolates, (underlined alginate phenotype), the only isolate that the phenotype on PIA did not match that on Blue/Chocolate agar; (*) mutations that have been previously described. (DOCX) [file pone.0208013.s001.docx]

| **S1 Table.** **General data of patients and bacterial isolates** | | | | |  |  |  |
| --- | --- | --- | --- | --- | --- | --- | --- |
| ***P. aeruginosa*** | **Patient's** | **PFGE** | **Alginate phenotype** | ***mucA* (64^#^)** | ***algT* (64^#^)** | ***mucB* (18^#^)** | ***mucD* (18^#^)** |
| **M/NM** | **age/gender*** |  |  |  |  |  |  |
| 1b M | 20/F | A | M (I) | Insertion GC at 583:Stop codon/Ala |  |  |  |
| 1b NM |  |  | NM (IV) | Insertion GC at 583:Stop codon/Ala |  |  | A409G:Ile/Val* |
| 2d M | 12/M | B | M (I) |  |  |  |  |
| 2d NM |  |  | NM (III) | G409T:Gly/Cys |  |  |  |
| 3h M | 42/M | C | M (I) | C35G:Ala/Gly, C364T:Pro/Ser, C367T:Stop codon* |  |  |  |
| 3h NM |  |  | NM (III) | C35G:Ala/Gly, C364T:Pro/Ser, C367T:Stop codon* |  |  |  |
| 7f M | 2/F | D | M (I) | C32T:Ser/Phe* |  |  |  |
| 7f NM |  |  | NM (III) |  | A508G:Thr/Ala |  |  |
| 9b NM | 13/F | E | NM (IV) |  |  | Insertion G at 41:Frameshift, ∆C57:Thr/His, G68A:Asp/Asn |  |
| 12 NM | 12/M | F | M (I) |  |  |  |  |
| 19a M | 29/M | G | M (I) | C382T:Stop codon* |  |  |  |
| 19a NM |  |  | NM (IV) | C382T:Stop codon* | A86G:Tyr/Cys | C611G:Ala/Gly, A631G:Thr/Ala | A409G:Ile/Val*, G1321A:Val/Ile* |
| 21a M | 27/F | J | M (I) | ∆C365:Frameshift, stop codon 385 |  |  |  |
| 21a NM |  |  | NM (III) | ∆C365:Frameshift, stop codon 385 |  |  |  |
| 22a M | 21/F | M | M (I) | ∆G168:Frameshift, stop codon 283 |  |  |  |
| 26d M | 10/F | I | M (I) | ∆G430:Frameshift, stop codon 439* |  |  |  |
| 26d NM |  | I1 | NM (IV) | ∆G430:Frameshift, stop codon 439* |  | C338T:Ser/Phe* |  |
| 27f NM | 2/M | H1 | NM (III) |  |  |  |  |
| 28f NM | 7/M | E2 | NM (III) |  |  |  |  |
| 33e NM | 16/F | G | NM (III) |  |  |  |  |
| 38l NM | 9/F | K | NM (III) |  |  |  |  |
| 40b M | 10/F | L | M (I) | ∆A568:Frameshift | A11G:Gln/Arg |  |  |
| 40b NM |  |  | NM (III) |  |  |  |  |
| 44 M | 12/M | N | M (I) | C349T:Stop codon* |  |  |  |
| 44 NM |  |  | NM (III) | C349T:Stop codon* | Insertion 9pb at 138:Frameshift* |  |  |
| 45 M | 20/M | α | M (I) | C349T:Stop codon* |  |  |  |
| 45 NM |  |  | NM (IV) |  |  | A631G:Thr/Ala*, C643T:Arg/Cys | G1321A:Val/Ile* |
| 47c M | 16/F | O | M (I) | ∆A578:Frameshift |  |  |  |
| 47c NM |  |  | NM (IV) | ∆A578:Frameshift | Insertion 9pb at 138:Frameshift* |  |  |
| 50e M | 22/F | λ | M (I) | ∆G430:Frameshift, stop codon 439* |  |  |  |
| 50e NM |  |  | NM (IV) | ∆G430:Frameshift, stop codon 439* |  |  |  |
| 58e NM | 13/F |  | NM (III) |  |  |  |  |
| 62a M | 26/M | P | M (I) | ∆G430:Frameshift, stop codon 439* |  |  |  |
| 62a NM |  |  | NM (IV) | ∆G430:Frameshift, stop codon 439* | A86G:Tyr/Cys |  |  |
| 64c M | 34/F | Q | M (I) | T45G:Asp/Glu |  |  |  |
| 64c NM |  |  | NM (IV) | T45G:Asp/Glu | ∆G157:Frameshift, stop codon 295 |  | A409G:Ile/Val* |
| 65e NM | 12/M | B | NM (III) |  |  |  |  |
| 71a NM | 6/M | R | NM (III) |  |  |  |  |
| 73c NM | 12/M |  | NM (IV) |  |  |  | A409G:Ile/Val* |
| 78a NM | 17/F | S | NM (III) |  |  |  |  |
| 79a M | 1/M | A | M (I) | Insertion GC at 583:Stop codon/Ala |  |  |  |
| 79a NM |  |  | NM (IV) | Insertion GC at 583:Stop codon/Ala | G49A:Gly/Arg |  | A409G:Ile/Val* |
| 84 M | 23/M | T | M (I) | C137T:Ala/Val*, ∆A437:Frameshift, stop codon 439 |  |  |  |
| 84 NM |  |  | NM (IV) | C137T:Ala/Val*, ∆A437:Frameshift, stop codon 439 | A86G:Tyr/Cys |  | A409G:Ile/Val* |
| 86e M | 10/M | π2 | M (I) | ∆G357:Frameshift, stop codon 385 |  |  |  |
| 86e NM |  |  | NM (IV) | ∆G357:Frameshift, stop codon 385 | ∆C556:Frameshift |  | G1321A:Val/Ile* |
| 88 NM | 20/M | U | NM (III) |  |  |  |  |
| 92 NM | 4/M | V | NM (III) |  |  |  |  |
| 95f M | 1/M | σ | M (I) | ∆C310:Frameshift, stop codon 385 |  |  |  |
| 95f NM |  |  | NM (IV) | ∆C310:Frameshift, stop codon 385 | C133T:Stop codon |  |  |
| 96 M | 31/F | W | M (I) | ∆G430:Frameshift, stop codon 439* |  |  |  |
| 96 NM |  |  | NM (III) | ∆G430:Frameshift, stop codon 439* |  |  |  |
| 103e M | 16/M | ψ | M (I) | C424T:Stop codon* |  |  |  |
| 104 M | 9/F | X | M (I) | Insertion C at 238:Frameshift, stop codon 478 |  |  |  |
| 104 NM |  |  | NM (IV) | Insertion C at 238:Frameshift, stop codon 478 |  |  | A409G:Ile/Val* |
| 114b M | 18/M | Y | M (I) | ∆G430:Frameshift, stop codon 439* |  |  |  |
| 114b NM |  |  | NM (III) | ∆G430:Frameshift, stop codon 439* | C528A:Phe/Leu |  |  |
| 116b M | 18/M | Z | M (I) | ∆C347:Frameshift, stop codon 385 |  |  |  |
| 116b NM |  |  | NM (III) | ∆C347:Frameshift, stop codon 385 | A176G:Tyr/Cys |  |  |
| 119 M | 45/M | δ | M (IV) | ∆G430:Frameshift, stop codon 439* |  | A631G:Thr/Ala*, C643T:Arg/Cys | C1310G:Thr/Ser, G1321A:Val/Ile* |
| 119 NM |  |  | NM (IV) | ∆G430:Frameshift, stop codon 439* |  | A631G:Thr/Ala*, C643T:Arg/Cys | C1310G:Thr/Ser, G1321A:Val/Ile* |
| 126 NM | 24/M | φ | NM (III) |  |  |  |  |
| 127 M | 8/F | θ | M (I) | C352T:Stop codon* |  |  |  |
| 127 NM |  |  | NM (IV) |  |  | ∆T733:Frameshift, stop codon 868 |  |

M: mucoid, NM: non-mucoid, age at first pulmonary secretion sample, gender M: male, F: female, ^#^ number of mucoid and non-mucoid isolates, (underlined alginate phenotype), the only isolate that the phenotype on PIA did not match that on Blue/Chocolate agar; (*) mutations that have been previously described.
